# Cross-cultural adaptation and psychometric validation of the Spanish version of the SBAR-LA rubric for structured communication in nursing simulation

**DOI:** 10.1371/journal.pone.0337533

**Published:** 2025-11-25

**Authors:** Jaime Carballedo-Pulido, Mariona Farrés-Tarafa, Juan Roldán-Merino, Marta Berenguer-Poblet, Montserrat Girabent-Farrés, Carla Otero-Arús, Susana Santos-Ruiz

**Affiliations:** 1 Campus Docent Sant Joan de Deu, Universitat de Vic-Universitat Central de Catalunya (UVIC-UCC), Sant Boi de Llobregat, Barcelona, Spain; 2 Department of Fundamental and Clinical Care Nursing, Hospitalet del Llobregat, Universitat de Barcelona, Campus de Bellvitge, Barcelona, Spain; 3 Simulation Group of the Spanish Society for Intensive Care and Coronary Unit Nursing (SEEIUC), Madrid, Spain; 4 Mental Health, Psychosocial and Complex Nursing Care Research Group, Barcelona, Spain; 5 Nursing Department, Campus Terres de l'Ebre, Universitat Rovira i Virgili, Tortosa, Spain; 6 Research Group on Advanced Nursing (CARING)-161, Universitat Rovira I Virgili, Tarragona, Spain; 7 Grupo DAFNiS, Campus Docent Sant Joan de Déu, Universitat de Barcelona, Sant Boi de Llobregat, Spain; 8 Parc Taulí Hospital Universitari. Institut d’Investigació i Innovació Parc Taulí (I3PT-CERCA). Universitat Autònoma de Barcelona, Sabadell, Spain; Universitat Autonoma de Barcelona, SPAIN

## Abstract

**Background:**

Although the SBAR framework is widely used in clinical and educational settings, there is a lack of validated Spanish-language tools that objectively assess its use by students in simulation. The adaptation and validation of the SBAR-LA rubric address this gap and provide a resource for training and evaluating structured communication.

**Objective:**

To conduct the cross-cultural adaptation and psychometric validation of the SBAR-LA rubric in Spanish for assessing structured communication skills in undergraduate nursing students during clinical simulation.

**Methods:**

A two-phase cross-sectional psychometric validation study was conducted. Phase one involved cross-cultural adaptation, including forward and backward translation, expert panel review, and cognitive debriefing with nursing students. Phase two assessed inter-rater reliability using Krippendorff’s alpha based on 97 performance evaluations obtained in different simulation scenarios. The SBAR-LA-Sp rubric contains 10 dichotomous items across the four SBAR dimensions.

**Results:**

The Spanish version of the SBAR-LA rubric demonstrated excellent inter-rater reliability, with a Krippendorff’s alpha of 0.933 (95% CI: 0.905–0.956). Internal consistency and agreement between raters were also high, confirming the instrument’s robustness.

**Conclusions:**

The Spanish version of the SBAR-LA rubric provides an objective measure of structured communication in nursing simulation. The findings support its use in academic training. Further research is needed to examine its effect on learning outcomes.

## Introduction

Effective information transfer, defined as the communication process through which healthcare professionals convey relevant clinical data and transfer responsibility for care, is a key factor in improving patient safety [1]. This process is a critical point in continuity of care due to its frequency and the associated risk of errors [2].

This process constitutes a critical point in continuity of care due to its frequency and the associated risk of errors [2]. Evidence indicates that a substantial proportion of adverse events are attributable to deficiencies in non-technical skills [3,4]. In Spain, the Ministry of Health reported that communication failures among healthcare professionals contribute to up to 70% of adverse events in clinical settings [5].

TeamSTEPPS®, a teamwork system developed in 2008 by the U.S. Department of Defense and the Agency for Healthcare Research and Quality, identified recurrent communication challenges within healthcare teams. These include delayed message delivery, absence of communication, inconsistent content, and unclear goals that impede timely clinical action [6]. Previous studies have also highlighted the hierarchical structure of healthcare teams as a major barrier to effective communication, as it limits open dialogue across professional ranks and compromises psychological safety [7,8]. Furthermore, differences in professional language may contribute to miscommunication: while physicians typically require specific diagnostic information, nurses may use broader, less precise terminology that does not always align with physicians’ expectations.

Nursing education should include core competencies related to patient safety, including the ability to communicate clinical information clearly, accurately, and in a structured manner. To support this, tools such as SBAR (Situation, Background, Assessment, Recommendation) have been developed to standardize information transfer among professionals. SBAR has shown effectiveness in improving clinical communication and reducing errors in various healthcare and educational settings [9–11]. The World Health Organization has identified structured communication as a strategic priority for patient safety [12]. SBAR also supports interprofessional communication by providing a shared language that reduces differences in communication styles and professional backgrounds [13,14].

Effective SBAR implementation requires both theoretical instruction and practical application. Clinical simulation is widely used to develop communication skills in healthcare students [15,16]. Simulated scenarios allow students to practice SBAR, support collaborative decision-making, and gain confidence in transferring critical information, which reduces the risk of communication errors [17–20]. Simulation also promotes critical thinking and reflective learning in a safe environment for learners and patients [21].

Several studies have demonstrated that simulation-based SBAR training significantly improves the quality of interprofessional communication among nursing students, fostering team cohesion and shared decision-making [22]. A recent study conducted among intensive care unit nurses found that those who received SBAR training during their undergraduate education demonstrated more effective use of the tool in clinical practice. These findings suggest that early exposure to structured communication models may result in long-term improvements in professional performance [23].

In undergraduate nursing education, the use of standardized and objective tools is essential for assessing students’ competency in structured communication using the SBAR model. Although some validated instruments, such as KidSIM, include communication as one component of teamwork evaluation [24–26], no available tools have been identified in the literature that specifically and exclusively assess structured information transfer. This underscores the need to translate and validate instruments capable of precisely measuring SBAR-related communication performance. Currently, the only available tool is the English-language SBAR-LA rubric.

The SBAR-LA (SBAR Brief Assessment Rubric for Learner Assessment) is a rubric designed to evaluate SBAR use in clinical simulation by nursing students. The original English version has demonstrated validity and reliability based on psychometric testing conducted in more than 200 simulation sessions with independent instructor evaluations. Reliability was assessed using internal consistency measures, including Krippendorff’s alpha for individual items and overall scores. Although the global assessment item showed lower reliability than expected, its strong correlation with the total analytical score supported the internal consistency of the instrument [25,26].

Currently, no validated Spanish-language version of the SBAR-LA rubric is available to accurately assess the impact of simulation-based training on structured communication competencies. Developing a new instrument is time- and resource-intensive, so the cross-cultural adaptation of existing tools offers a practical and efficient alternative. This approach preserves the psychometric integrity of the original instrument, reduces research costs, and enables meaningful comparisons with national and international studies using the same tool [27,28]. In this context, the objective of this study is to conduct the cross-cultural adaptation and initial psychometric validation of the SBAR-LA instrument for use in the Spanish context, assessing its inter-rater reliability during clinical simulation.

## Methods

### Study design

This prospective observational study employed a psychometric approach and was conducted in two phases. The first phase involved the cross-cultural adaptation of the SBAR-LA instrument into Spanish and the evaluation of content validity by a panel of expert nurses with clinical simulation expertise. In the second phase, the reliability of the adapted instrument was examined, followed by a descriptive cross-sectional analysis.

### Study setting

The study was conducted at two Spanish universities offering undergraduate nursing programs, both of which formally integrate simulation-based training into their curricula.

### Sample and recruitment

The target population consisted of third-year undergraduate nursing students of both sexes enrolled during the 2023–2024 academic year at the participating institutions. Eligibility criteria included prior completion of second-year clinical placements and enrollment in a mandatory clinical simulation module. A non-probability convenience sampling method was used.

Participants were assigned to simulation sessions focusing on clinical handoff communication. In each session, students were randomly selected using a blind draw with colored balls. Students who selected a green ball were assigned active roles in the simulation and were subsequently evaluated using the Spanish-adapted SBAR-LA instrument. Two independent raters, not involved in the simulation facilitation or study design, conducted the assessments to ensure objectivity.

Students who were absent on the day of their assigned simulation session were excluded. A total of 97 valid performance assessments were collected across various clinical scenarios. This sample size was deemed adequate in accordance with Streiner’s recommendation of 5–20 participants per item for psychometric validation [29].

### Procedure

#### Phase 1: translation and adaptation process.

The first phase followed a five-step process to ensure the linguistic and conceptual equivalence of the SBAR-LA instrument in Spanish.

1. **Forward Translation:** Two bilingual nurses, native Spanish speakers, independently translated the original English version of the SBAR-LA instrument into Spanish. Both received the original instrument along with a brief description of its purpose and characteristics. This step yielded two initial Spanish versions (T1 and T2).
2. **Expert Committee Review:** A review panel (n = 6) was convened, comprising three faculty members with expertise in clinical simulation, the two translators, and the principal investigator. The panel evaluated the semantic equivalence of T1 and T2, and through discussion and consensus, produced a single unified version (V1).
3. **Back-Translation:** Two native English-speaking translators, blind to the original version but informed about the purpose of the instrument, independently translated V1 back into English, resulting in two back-translated versions (RT1 and RT2).
4. **Final Review and Validation:** Based on RT1 and RT2, the expert committee produced a revised Spanish version (V2) and compared it to the original English instrument to ensure semantic, linguistic, and conceptual equivalence. This process led to the final Spanish version.
5. **Usability Testing:** The final version was pilot tested with 10 undergraduate nursing students to assess comprehension, ease of use, and estimated completion time. Based on participant feedback, minor revisions were made, and the definitive version, named SBAR-LA-Sp, was finalized.

In addition to administering the SBAR-LA-Sp instrument, demographic data including age and sex were collected for each participant.

### Data collection and variables

The SBAR (Situation, Background, Assessment, Recommendation) technique, developed by the Institute for Healthcare Improvement (IHI), provides a standardized framework to enhance communication among healthcare professionals. This structured approach facilitates the clear and concise exchange of critical information in clinical settings.

In this study, the SBAR-LA-Sp instrument, a Spanish adaptation of the SBAR Brief Assessment Rubric for Learner Assessment (SBAR-LA), was utilized to evaluate the effectiveness of structured communication during clinical simulations. The instrument comprises 10 dichotomously scored items (0 = skill not demonstrated; 1 = skill demonstrated), distributed across the four SBAR dimensions:

In this study, the SBAR-LA-Sp instrument, a Spanish adaptation of the SBAR Brief Assessment Rubric for Learner Assessment (SBAR-LA), was utilized to evaluate the effectiveness of structured communication during clinical simulations. The instrument comprises 10 dichotomously scored items (0 = skill not demonstrated; 1 = skill demonstrated), distributed across the four SBAR dimensions:

**Situation (Items 1–4):** Assessment of the ability to clearly describe the current clinical situation.

**Background (Items 5–7):** Evaluation of the provision of relevant patient history and context.

**Assessment (Item 8):** Appraisal of clinical reasoning and interpretation.

**Recommendation (Items 9–10):** Examination of the formulation of appropriate clinical suggestions.

The total score reflects overall proficiency in structured communication, with higher scores indicating greater adherence to the SBAR methodology. To ensure objectivity, two independent evaluators, not involved in the simulation facilitation, assessed each participant’s performance using the SBAR-LA-Sp instrument.

### Data analysis

Inter-rater reliability was assessed using Krippendorff’s alpha coefficient, calculated with a 95% confidence interval. The following thresholds were applied for interpretation: values ≥ 0.800 indicate excellent reliability; values between 0.60 and 0.79 suggest good reliability; values between 0.40 and 0.59 denote moderate reliability; and values < 0.400 reflect poor reliability [30]. All statistical analyses were performed using IBM SPSS Statistics for Windows, Version 29.0 (IBM Corp., Armonk, NY).

### Ethical considerations

This study was approved by the Research Ethics Committee of Campus Docent Sant Joan de Déu (Approval Code: Pr2/23). All participants received both oral and written information regarding the study’s objectives and the confidentiality of their data. Written informed consent was obtained from all students prior to their participation. Only those students present during the study briefing and who voluntarily agreed to participate were included in the study.

## Results

All items were translated and back translated without significant difficulties, and there was no need to modify the original format of the scale. To achieve the highest possible degree of semantic, idiomatic, and conceptual equivalence, the expert committee made modifications to specific items:

**Item 1:** Modified to include the patient’s name and professional category.

**Item 2:** Adjusted to require full name (first and last), without the option to include partial information.

**Item 3:** Gender was removed to avoid potential gender bias.

**Item 6:** Changed from “e.g., neurological status, lab values, vital signs, patient demands” to “e.g., age, allergies, medical history, regular medications, recent events, complementary tests.”

**Item 7:** Reworded from “Does not provide unnecessary information” to “Only provides necessary information” to avoid a double negative in the phrasing.

**Item 8:** Emphasis was placed on “summary” to clarify that the item assesses the ability to summarize the relevant clinical problem rather than performing a complete A–E assessment.

**Item 8** (continued): The example referring to warfarin was removed as it pertained to a very specific case.

**Items 9 and 10:** These were restructured, with the second example originally in item 9 being reassigned to item 10.

These changes are detailed in Table 1, which presents the semantic equivalence between the original English and the adapted Spanish versions of the items.

**Table 1:** Semantic equivalence of items from English to Spanish after psychometric Validation.

Item	English		Spanish	
	SBAR Category: Situation		SBAR Categoría: Situación	
	Sub-category	Description	Sub-categoría	Descripción
**Item 1**	Identifies self	Uses name for full credit	Se identifica a si mismo	Utiliza el nombre y la categoría para la puntuación máxima
**Item 2**	Provides patient name	First, last, or both	Proporciona el nombre del paciente	Nombre y apellidos
**Item 3**	Provides a second patient identifier	, DOB, age, gender, room number	Proporciona un segundo identificador del paciente	Ej., fecha de nacimiento, edad, número de habitación
**Item 4**	Expresses situation, issue, concern	To get full credit student needs to specifically state that “I need to speak to you”, state “urgency,” or “cannot wait”	Expresa la situación, el problema o la preocupación	Para obtener la puntuación máxima, el estudiante debe indicar específicamente que “necesito hablar con usted”, indicar “urgencia” o “no puede esperar”
	**SBAR Category: Background**		**SBAR Categoría: Antecedentes**	
**Item 5**	States the context	“Are you aware of...” patient condition, reason for admission, and/or change in status	Expone el contexto	“¿Está usted al corriente de...?” estado del paciente, motivo del ingreso y/o cambio de estado
**Item 6**	States recent findings	, mental status, lab values, vital signs, patient complaints	Expone los resultados recientes	Ej., Edad, alergias, antecedentes patológicos, medicación habitual Sucesos recientes Pruebas complementarias realizadas
**Item 7**	Provides facts only	Does not provide unnecessary information	Proporciona solo hechos	Solo proporciona información necesaria
	**SBAR Category: Assessment**		**SBAR Categoría: Evaluación**	
**Item 8**	Provides summary assessment of problem	Need a summary of the underlying problem/concern which in this case is acute change in mental status and new information regarding coumadin	Proporciona un **resumen** de la evaluación del problema	Expone un resumen del problema/preocupación subyacente.
	**SBAR Category: Recommendation**		**SBAR Categoría: Recomendación**	
**Item 9**	Provides concrete suggested action	“Based on this assessment, I request/recommend that... i.e., come in and evaluate patient, get CT earlier.” For full credit also needs to include clear statement on confirmation of plan, i.e., “Are you coming in, how do you want to be reached with new result.”	Proporciona sugerencias sobre acciones concretas	“Basándome en esta evaluación, solicito/recomiendo que... ej., que venga a evaluar al paciente, que se realce un TAC pronto”.
**Item 10**	Provides contact information	Phone number, texting app, pager	Proporciona información de cómo se retoma el contacto	“¿Vendrás a valorar al paciente?,¿cómo quiere que se le informe de los nuevos resultados?”. Número de teléfono, aplicación de mensajería, busca

This is a Spanish adaptation of the original SBAR-LA rubric developed by Davis et al. (2021), published in MedEdPORTAL under a CC BY 4.0 license. Available at: https://doi.org/10.15766/mep_2374-8265.11184. The adaptation was performed for validation purposes in undergraduate nursing students.

Additionally, specific instructions were added for instructors and evaluators to standardize the completion criteria and ensure consistent scoring.

Once the semantically adapted version was finalized, a pilot test was conducted with a sample of 10 nursing students and two independent raters. Both raters agreed that the tool was easy to use and feasible to complete during the simulation scenario. They also evaluated the completion instructions positively, noting their usefulness in guiding the assessment process.

This section may be divided by subheadings. It should provide a concise and precise description of the experimental results, their interpretation, as well as the experimental conclusions that can be drawn.

### Reliability

The nursing students who participated in the simulation had a mean age of 22.8 years (SD = 3.8). The majority of participants were female, representing 86.2% of the sample.

As shown in Table 2, the Krippendorff’s alpha reliability coefficient obtained for the total score of the 10-item scale was 0.933, with a 95% confidence interval ranging from 0.905 to 0.956, indicating excellent reliability.

**Table 2:** Reliability analysis of the SBAR-LA scale using Krippendorff’s Alpha.

SBAR Category	Items	Krippendorff’s alpha	Lower IC	Upper IC
**Situation**	1) Identifies self	0,907	0,814	0,977
	2) Provides patient name	0,923	0,819	1,000
	3) Provides a second patient identifier	0,807	0,679	0,914
	4) Expresses situation, issue, concern	0,767	0,601	0,934
**Background**	5) States the context	0,746	0,564	0,891
	6) States recent findings	0,847	0,719	0,949
	7) Provides facts only	0,686	0,451	0,882
**Assessment**	8) Provides summary assessment of problem	0,805	0,642	0,935
**Recommendation**	9) Provides concrete suggested action	0,892	0,784	0,978
	10) Provides contact information	0,902	0,804	0,976
**Total Situation**		0,748	0,636	0,846
**Total Background**		0,731	0,605	0,853
**Total Assessment**		0,805	0,642	0,935
**Total Recommendation**		0,918	0,822	0,982
**Total Scale**		**0,933**	**0,905**	**0,956**

**Fig 1:**
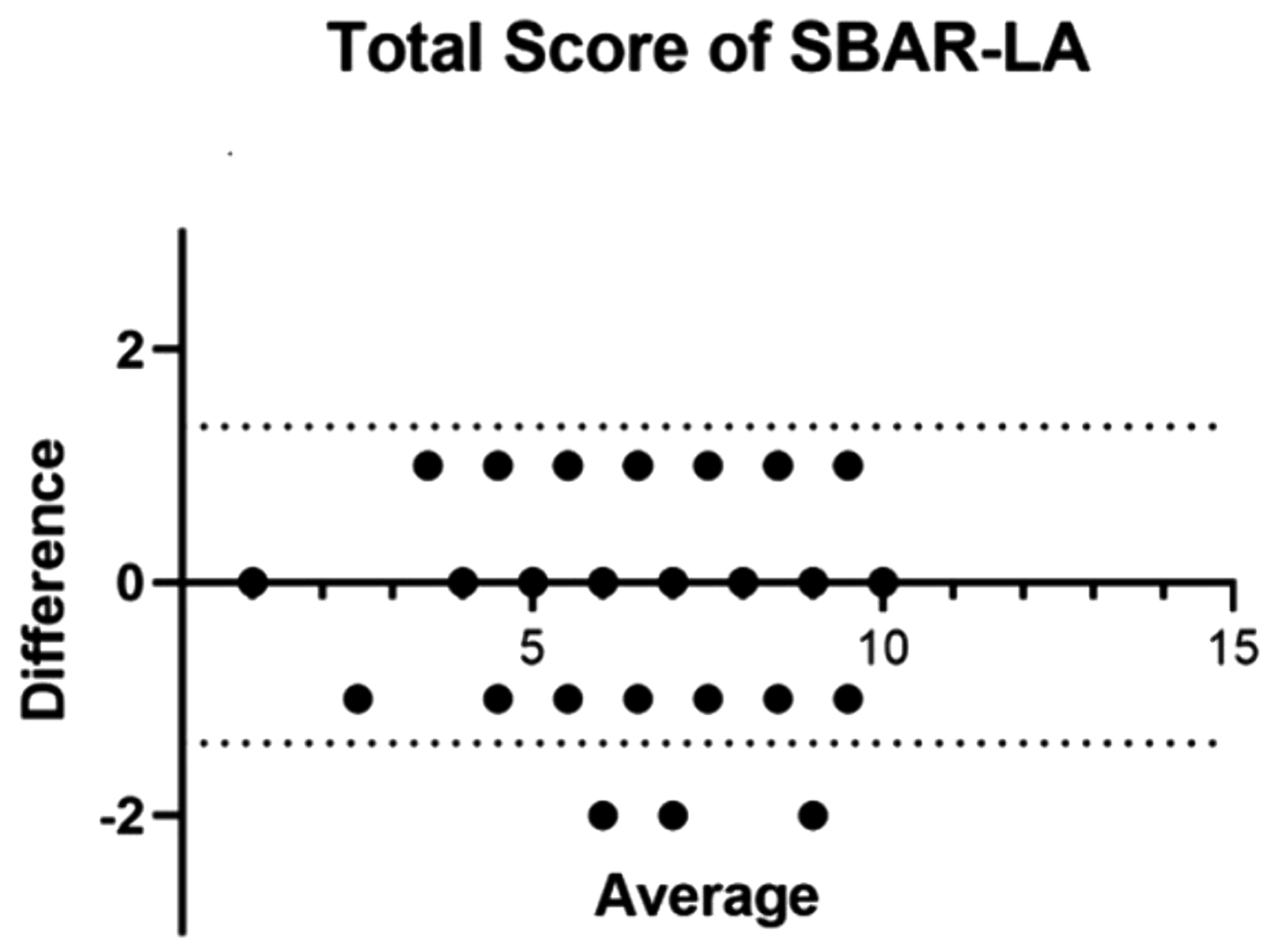
Bland–Altman plot for the total score of the SBAR-LA-Sp instrument.

Fig 1 presents the Bland–Altman analysis for the total score of the SBAR-LA scale, showing that the vast majority of data points fall within the confidence interval, which suggests adequate measurement stability.

## Discussion

This study facilitated the cross-cultural adaptation and validation of the SBAR-LA instrument for assessing information transfer during clinical simulation scenarios involving nursing students. The findings confirm the instrument’s high reliability and its applicability within the Spanish educational context, reinforcing its potential as a valuable tool for evaluating and enhancing structured communication skills in nursing education.

The Krippendorff’s alpha coefficient obtained for the total scale score was 0.933, indicating excellent reliability and exceeding commonly accepted thresholds for assessment tools. These results align with those reported in the original validation study of the English version of the instrument [27], supporting the validity of the cross-cultural adaptation conducted in this research.

Regarding item-level reliability, all items except one demonstrated good or excellent inter-rater agreement. Item 7 (“Provides facts only”) showed lower and less stable reliability (α = 0.686; 95% CI: 0.451–0.882), indicating variability in scoring. This may reflect a broader interpretive scope or differences in evaluator expectations. These findings suggest the need to refine the scoring criteria or provide additional rater training to ensure consistent application in future use. Similarly, the item “Provides context” showed good reliability; however, its confidence interval ranged from moderate to very good, indicating some degree of uncertainty in the estimate.

The reliability patterns observed in this study align with findings reported in other contexts, such as rehabilitation settings [31]. Analysis of the four SBAR dimensions (Situation, Background, Assessment, and Recommendation) revealed that three dimensions demonstrated good reliability, while the Recommendation dimension exhibited excellent reliability, with a confidence interval ranging from 0.822 to 0.982, underscoring its internal consistency.

Collectively, these results support the SBAR-LA instrument as a psychometrically robust tool for evaluating structured communication in clinical simulation settings. Future research should focus on revising items with less stable reliability estimates to further enhance the instrument’s accuracy and utility in nursing education.

The significance of structured communication tools like SBAR-LA has been emphasized in previous studies assessing the effectiveness of SBAR in improving interprofessional communication [22,23]. Research indicates that SBAR facilitates the structured transmission of clinical information, reduces communication errors, and enhances patient safety in both clinical and educational environments [18,27].

The present study reinforces these findings by providing a reliable and valid psychometric instrument for assessing nursing students’ competencies in structured communication techniques during simulation-based training. Furthermore, the adaptation of SBAR-LA into Spanish ensures its applicability in both academic and clinical settings across Spanish-speaking contexts, enabling nursing students to develop standardized communication skills that support effective interprofessional collaboration.

However, the contribution of SBAR-LA-Sp to learning outcomes and communication skill development should be understood as indirect. The instrument supports structured practice and provides consistent feedback during simulations, which can facilitate the acquisition and consolidation of communication competencies. With repeated use, students may internalize the SBAR structure and transfer it to real clinical situations, potentially influencing long-term professional performance. Nevertheless, this study did not directly measure learning outcomes or real-world clinical performance, and therefore, these effects remain hypothetical and should be examined in future longitudinal research.

This cross-cultural adaptation also contributes to consistency in competency assessment within national contexts by providing a standardized tool that allows for comparison with international studies. The availability of validated versions in multiple languages supports the global standardization of clinical communication protocols, promoting safer and more effective practices in nursing education and patient care [27].

The use of dichotomous scoring aligns with the original structure of the SBAR-LA instrument, ensuring methodological equivalence throughout the cross-cultural adaptation process. While this format facilitates real-time evaluation during simulation, it may limit sensitivity to nuanced performance or intermediate levels of competence.

Although SBAR-LA is conceptually linked to patient safety and interprofessional collaboration, these aspects were not directly assessed in this study. Therefore, any claims regarding its impact should be made cautiously and within the context of complementary research.

Future research should explore the integration of SBAR-LA into nursing curricula and evaluate its long-term impact on clinical performance, patient safety and quality of care in real clinical world settings.

### Limitations

This study has several limitations. Firstly, the use of a non-probabilistic convenience sample may restrict the generalizability of the findings. However, the sample size was adequate for psychometric validation, providing robust reliability estimates.

A more diverse and geographically broader sample would strengthen external validity and allow the results to be extrapolated with greater confidence to different educational contexts.

Secondly, the study was conducted within a specific educational setting, which may limit its applicability to other nursing programs. Nevertheless, the rigorous cross-cultural adaptation process enhances the instrument’s relevance in Spanish-speaking contexts.

While some individual items exhibited variability in their reliability estimates, particularly item 7, the overall internal consistency was excellent, reinforcing the instrument’s psychometric strength. Additionally, the use of simulation-based validation, although not fully replicating real clinical environments, offers a controlled and safe setting to assess students’ communication skills without compromising patient safety.

Finally, the absence of longitudinal follow-up in this study limits the ability to assess the long-term impact of SBAR-LA training on clinical performance and patient safety. Future research should address this gap to provide a more comprehensive evaluation of the instrument’s effectiveness over time.

### Relevance for nursing education

Clinical simulation is widely recognized as an effective methodology for developing both technical and non-technical skills, including communication and teamwork. Integrating the SBAR-LA-Sp instrument into simulation-based training provides a standardized framework for evaluating these competencies, fostering a culture of patient safety from the early stages of professional education. Given that communication errors remain a leading cause of adverse events in healthcare settings, the use of validated tools like SBAR-LA enhances the objective assessment of students’ ability to transfer critical information, thereby supporting interprofessional collaboration and promoting patient safety.

The Spanish version of SBAR-LA shows strong potential for use in educational settings across Spanish-speaking countries. However, cultural validation in other regions is necessary to ensure semantic and functional equivalence of the instrument, taking into account the linguistic and pedagogical particularities of each context.

### Implications for practice and research

This study opens new avenues in nursing education and research. The Spanish version of SBAR-LA can be integrated into simulation programs to objectively measure communication and information transfer skills. Future research should focus on evaluating the instrument’s applicability in real clinical environments and its impact on reducing communication-related errors. Additionally, conducting studies across various Spanish-speaking countries would help expand the external validity of the instrument.

Longitudinal studies are also needed to assess the long-term impact of SBAR-LA training on students’ clinical performance and patient safety. Continuous improvement of the instrument, particularly in items with less stable reliability estimates, will further enhance its effectiveness as an educational and assessment tool in nursing training.

Additionally, validating the instrument in other Spanish-speaking regions will help ensure semantic and functional equivalence across cultural and educational contexts, facilitating its broader implementation.

## Conclusions

The Spanish version of the SBAR-LA-Sp instrument shows strong inter-rater reliability and semantic equivalence with the original version, supporting its use in clinical simulation contexts. Its use allows for standardized assessment of structured communication competencies and contributes to patient safety training in early stages of nursing education. While the study has methodological limitations, the findings provide a foundation for integrating SBAR-LA-Sp into nursing curricula. Further cultural validation in other Spanish-speaking contexts and longitudinal studies in real clinical environments are recommended to strengthen its evidence base.

## Supporting information

S1 Data (XLSX)
